# Investigation of predictability and influence factors of the achieved lenticule thickness in small incision lenticule extraction

**DOI:** 10.1186/s12886-020-01374-4

**Published:** 2020-03-17

**Authors:** Fang Wu, Houfa Yin, Xinyi Chen, Yabo Yang

**Affiliations:** Eye Center, Second Affiliated Hospital, College of Medicine, Zhejiang University, Jie Fang Road 88#, Hangzhou, 310009 People’s Republic of China

**Keywords:** SMILE, Lenticule thickness, Predictability, SE, Lenticule depth

## Abstract

**Background:**

To evaluate the differences between the predicted and achieved lenticule thickness (ΔLT) after small incision lenticule extraction (SMILE) surgery and investigate relationships between ΔLT and predicted lenticule thickness in SMILE.

**Methods:**

A total of 184 eyes from 184 consecutive patients who underwent SMILE were included in this prospective study. One eye for each patient was randomly selected and included for statistical analysis. To achieve emmetropia, nomogram adds 10% correction of spherical refractive. An ultrasound pachymetry measurement and Scheimpflug camera corneal topography were obtained before and at 3 months after SMILE. The achieved lenticule thickness was calculated by comparing the preoperative examinations with postoperative examinations using ultrasound pachymetry and Pentacam software measurements. The pupil center and corneal vertex were selected as the 2 locations for measurement calculation on Pentacam. Analysis of variance (ANOVA) was performed to compare mean pachymetry values using different instruments. Linear regression analyses were performed between the VisuMax readout lenticule thicknesses and the measured maximum corneal change, between ΔLT and predicted lenticule thickness.

**Results:**

On average, the achieved lenticule thickness measured with ultrasound pachymetry was 13.02 ± 8.87 μm thinner than the predicted lenticule thickness. The proportion of ΔLT in predicted values is 11.9% (ultrasound) and about 15% (Pentacam). Linear regression analysis showed significant relationships between the predicted and each achieved lenticule thickness. Each ΔLT was significantly related to predicted lenticule thickness (ultrasound: *R*^2^ = 0.242; pupil center from Pentacam: *R*^2^ = 0.230).

**Conclusions:**

An overestimation of achieved lenticule thickness was evident in this study which may exclude eligible SMILE patient. Also, our results showed that 10% increase of spherical refractive correction in the nomogram is appropriate. Furthermore, clinicians should subtract 10% of the predicted lenticule thickness to calculate the residual corneal stroma bed thickness.

## Background

Small incision lenticule extraction (SMILE) was first described by Sekundo et al. and Shah et al. in 2011 for the treatment of myopia and myopic astigmatism [1, 2]. Compared with Excimer laser surgery, SMILE is an “all-in-one” surgery that involves the creation of an intrastromal lenticule and a peripheral incision in one step using a femtosecond laser, and manual extraction of the lenticule later. In this way, SMILE surgery avoids or minimizes errors associated with excimer laser ablation, such as stromal hydration [3], laser fluence [4–6], environmental temperature, and relative humidity [7]. Therefore, the thickness of the intrastromal lenticule created at the beginning of the surgery determines the safety and accuracy of SMILE surgery. A close consistency would be expected between the predicted and achieved lenticule thickness. However, previous studies have reported that there was still a difference between the predicted and achieved lenticule thickness (ΔLT). Reinstein et al. [8] detected a systematic overestimation of central lenticule thickness of approximately 8 μm. Luft et al. [9] also found that the predicted lenticule thickness was thicker than the achieved lenticule thickness, especially with higher myopic correction.

Ultrasound pachymetry has been the gold standard in measuring corneal thickness. Scheimpflug imaging [10], as a new method, also allows the measurement of corneal thickness. The principle of Scheimpflug imaging uses optical sectioning of the cornea with maximum depth of focus [11].

In the current study, we included a large number of patients to investigate the predictability between the VisuMax readout and achieved lenticule thickness measured at 3 months postoperatively. The aim of this study was to assess the ΔLT in SMILE using ultrasound pachymetry and Scheimpflug imaging and to investigate the associations of measured lenticule thickness with prediction on VisuMax, and ΔLT with predicted lenticule thickness.

## Methods

This prospective study included 184 consecutive patients who underwent a SMILE procedure at the Eye Center, Second Affiliated Hospital, College of Medicine, Zhejiang University, from November 2017 to August 2018. This study followed the tenets of the Declaration of Helsinki and was approved by the Ethics Committee of the Second Affiliated Hospital of Zhejiang University. Written informed consent was obtained from the subjects before participating in this study. Patients with the ocular pathology (eg, keratoconus) or a history of ocular surgery or trauma were excluded from participation in the study.

Each patient underwent an ophthalmologic examination, including manifest refraction spherical equivalent (MRSE), slit-lamp examination, ultrasound pachymetry measurement (Tomey SP-3000 pachymeter, Nagoya, Aichi-ken, Japan) and Pentacam imaging (Oculus Optikgeräte GmbH, Wetzlar, Germany). Ultrasound pachymetry and Pentacam scanning were performed preoperatively and at the 3-month follow-up.

### Surgical procedure

All SMILE procedures were performed by the same surgeon (YYB) using the VisuMax femtosecond laser system (Carl Zeiss Meditec AG, Jena, Germany). The routine procedures of the SMILE surgery have been described in a previous study [12]. In this study, the laser cut energy index was 155 nJ; the intended cap thickness was 110 μm to 140 μm; the programmed optical zone diameter was between 6.1 and 6.5 mm; and the diameter of the cap was 1 mm larger than the diameter of the lenticule. The optical zone and cap thickness were selected on the basis of the pupil diameter and percent tissue alert (PTA). A recommended nomogram adjustment was implemented for all subjects. To achieve emmetropia, the nomogram adds 10% correction of spherical refractive, as suggested by the manufacturer and as is the similar experience of other surgeons [13, 14]. The predicted lenticule thickness, following the nomogram adjustment, was displayed by the VisuMax software and recorded for statistical analysis.

### Postoperative treatment regimen

Patients were instructed to wear plastic shields for 7 nights. The Levofloxacin eye drops (Cravit; Santen Pharmaceutical Co Ltd., Osaka, Japan) and 0.1% Fluorometholone eye drops (Santen Pharmaceutical Co Ltd., Osaka, Japan) were prescribed 4 times daily for 1 and 2 weeks, respectively. Preservative-free artificial tears were prescribed 4 times a day for a month. The patients were followed up at 1 day, 1 week and 1 and 3 months. Pentacam scanning and ultrasound pachymetry were performed at the 3-month postoperative visit.

### Achieved Lenticule thickness calculation

The achieved lenticule thickness data were calculated by comparing the pre- and postoperative examinations with Pentacam software and ultrasound pachymetry measurement, respectively. Noncontact assessment (Pentacam) was consistently performed first. The rotating Pentacam Scheimpflug camera measures corneal thickness normal to the anterior surface tangent [15]. The pachymetry values were provided at 3 points [16], including the corneal vertex, pupil center and thinnest point. During the examination, the automatic release mode was used [17]. In this study, the intended treatment center was the corneal vertex. Since the position of the thinnest point of cornea varies greatly from person to person. The corneal vertex and pupil center were selected as the two locations to calculate the achieved lenticule thickness.

When corneal thicknesses were measured by ultrasound pachymetry, all patients underwent topical anesthesia using proparacaine 0.5% (Alcaine; Alcon-Couvreur n.v., Puurs, Belgium), and an average of 10 consecutive measurements was obtained in each eye. For ultrasound pachymetry measurements, a default velocity of 1640 m/s was used.

### Statistical analysis

In our study, only one eye for each patient was randomly selected and included for statistical analysis to ensure that the measurements from eyes can be treated as independent [18]. All statistical analyses were performed using the SPSS software package, version 16.0 (SPSS Inc., IBM, USA). The Kolmogorov-Smirnov normality test was used to assess the normal distribution of data. One-way analysis of variance (ANOVA) with post hoc Bonferroni test was performed to compare mean pachymetry values using different instruments, both preoperatively and postoperatively. Agreement was evaluated using Bland-Altman charts, in which the central corneal thickness and achieved lenticule thickness between the measurements are plotted against their mean [19]. The 95% limits of agreements (LoA) of the bias were calculated as the mean ± 1.96 standard deviations. Linear regression analysis was performed, and the coefficient of determination (R^2^) was calculated to investigate the correlation between the predicted and achieved lenticule thickness, between the predicted lenticule thickness and ΔLT. *P* values less than 0.05 were considered statistically significant.

## Results

A total of 184 myopic eyes of 184 patients (93 men and 91 women) with a mean age of 24.24 ± 6.28 years (range: 18 to 42 years) who underwent SMILE surgery were analyzed preoperatively and 3 months postoperatively. No intraoperative or postoperative complications were encountered during the follow-up. The mean attempted preoperative spherical equivalent (SE) refractive error was − 5.34 ± 1.63 diopters (D) (range: − 9.38 to − 1.5 D). The mean SE of the surgical refractive correction (with nomogram adjusted) was − 5.85 ± 1.79 D (range: − 10.2 to − 1.65 D). The mean preoperative and treated cylinder errors were both − 0.65 ± 0.57 D (range: − 2.75 to 0 D).

The programmed optical zone was selected on the basis of the pupil diameter and PTA. The optical zone diameter was 6.1 mm in 3 eyes (1.6%), 6.2 mm in 2 eyes (1.1%), 6.3 mm in 4 eyes (2.2%), 6.4 mm in 10 eyes (5.4%), and 6.5 mm in 165 eyes (89.7%). The programmed cap thickness was 110 μm in 24 eyes (13.0%), 120 μm in 87 eyes (47.3%), 130 μm in 61 eyes (33.2%), 135 μm in 6 eyes (3.3%), and 140 μm in 7 eyes (3.8%). The programmed mean minimum thickness at the edge of the lenticule was 11.88 ± 3. 07 μm (range: 10 to 30 μm).

### Predictability and stability

**Fig. 1:**
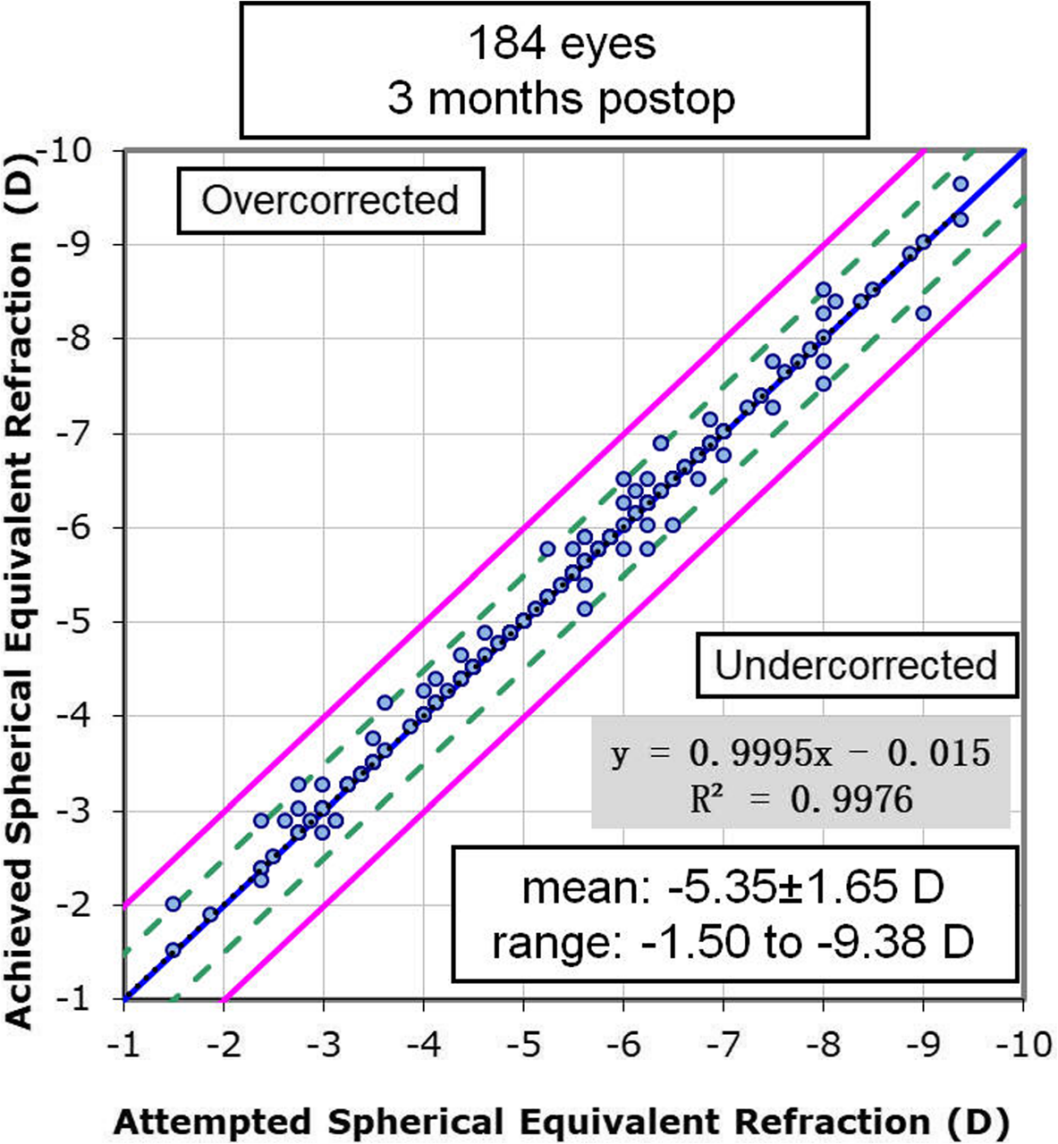
Achieved versus attempted changes in spherical equivalent at 3 months follow-up for 184 patients

**Fig. 2:**
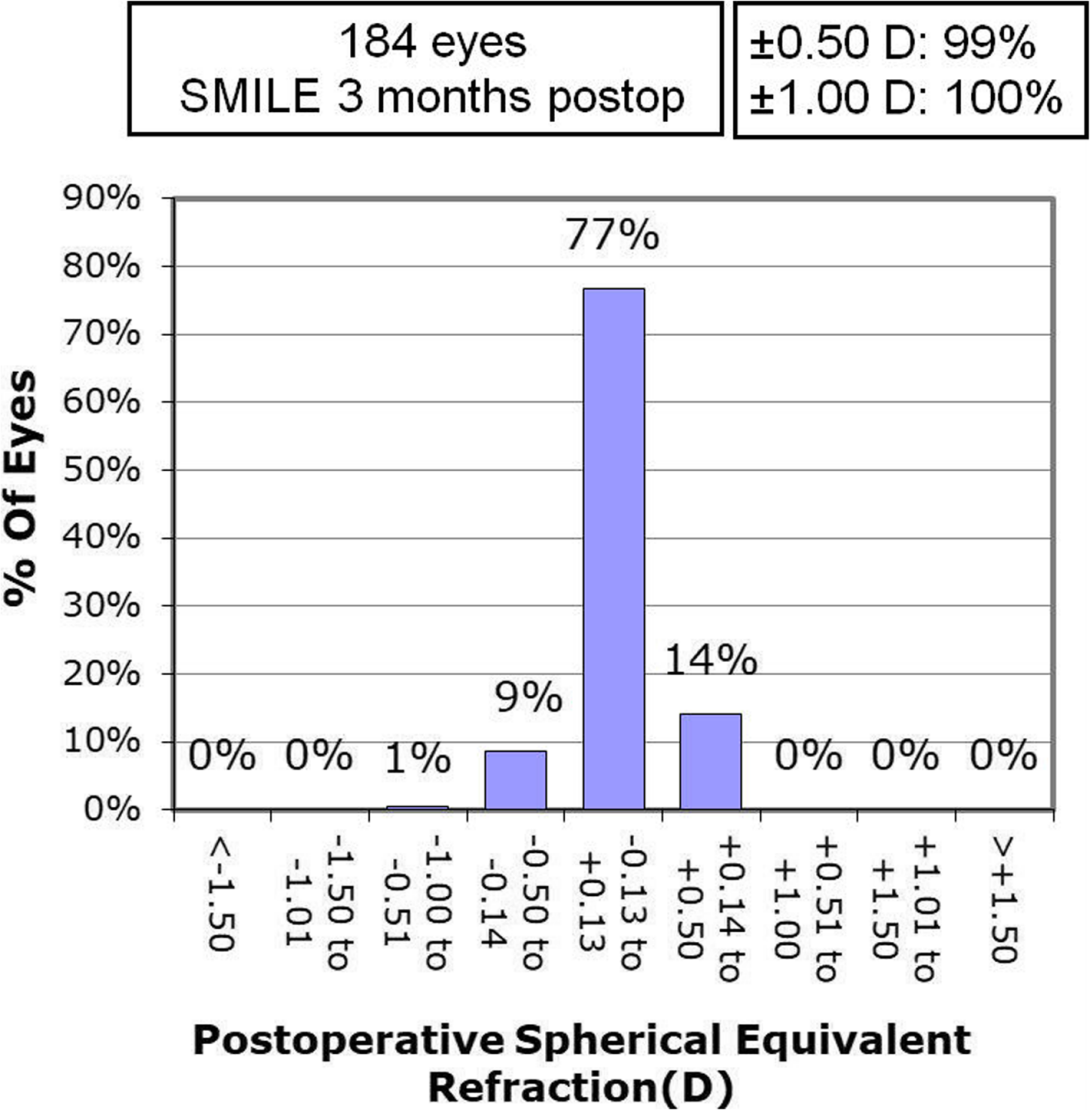
Accuracy of spherical equivalent refraction for 184 patients at 3 months after SMILE surgery

At 3 months postoperatively, the achieved SE was − 5.36 ± 1.61 D, which is significantly correlated with the attempted SE. The linear regression analysis of attempted SE versus achieved SE refraction at 3 months after SMILE is shown in Fig. 1. The uncorrected distance visual acuity (UDVA) was 20/20 or better in 178 eyes (96.7%). All of the eyes (100%) that underwent SMILE surgery had a postoperative UDVA of 20/30 or better. The predictability of SMILE surgery at 3 months postoperatively is displayed in Fig. 2. At the 3 month follow-up, 99% (183) of the eyes and 100% (184) of eyes were within ±0.5 and ± 1.0 D. The postoperative SE was 0.02 ± 0.17 D (range: − 0.75 to 0.5 D).

### Assessment corneal thickness

**Fig. 3:**
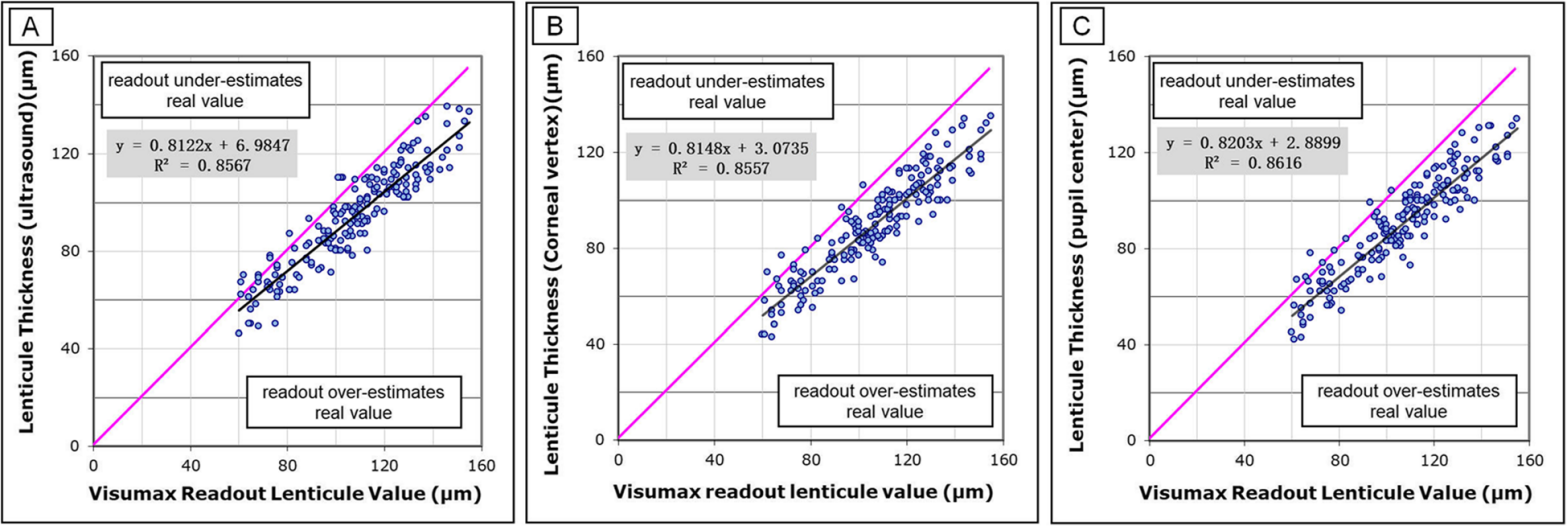
Correlation between the predicted and achieved lenticule thickness measured with ultrasound pachymetry. **a**. Correlations between predicted and achieved lenticule thickness measured with Pentacam software at the corneal vertex (**b**) and the pupil center (**c**). The regression equations and coefficients of determination (R^2^) are displayed. The red dotted line indicates a slope of 1

**Fig. 4:**
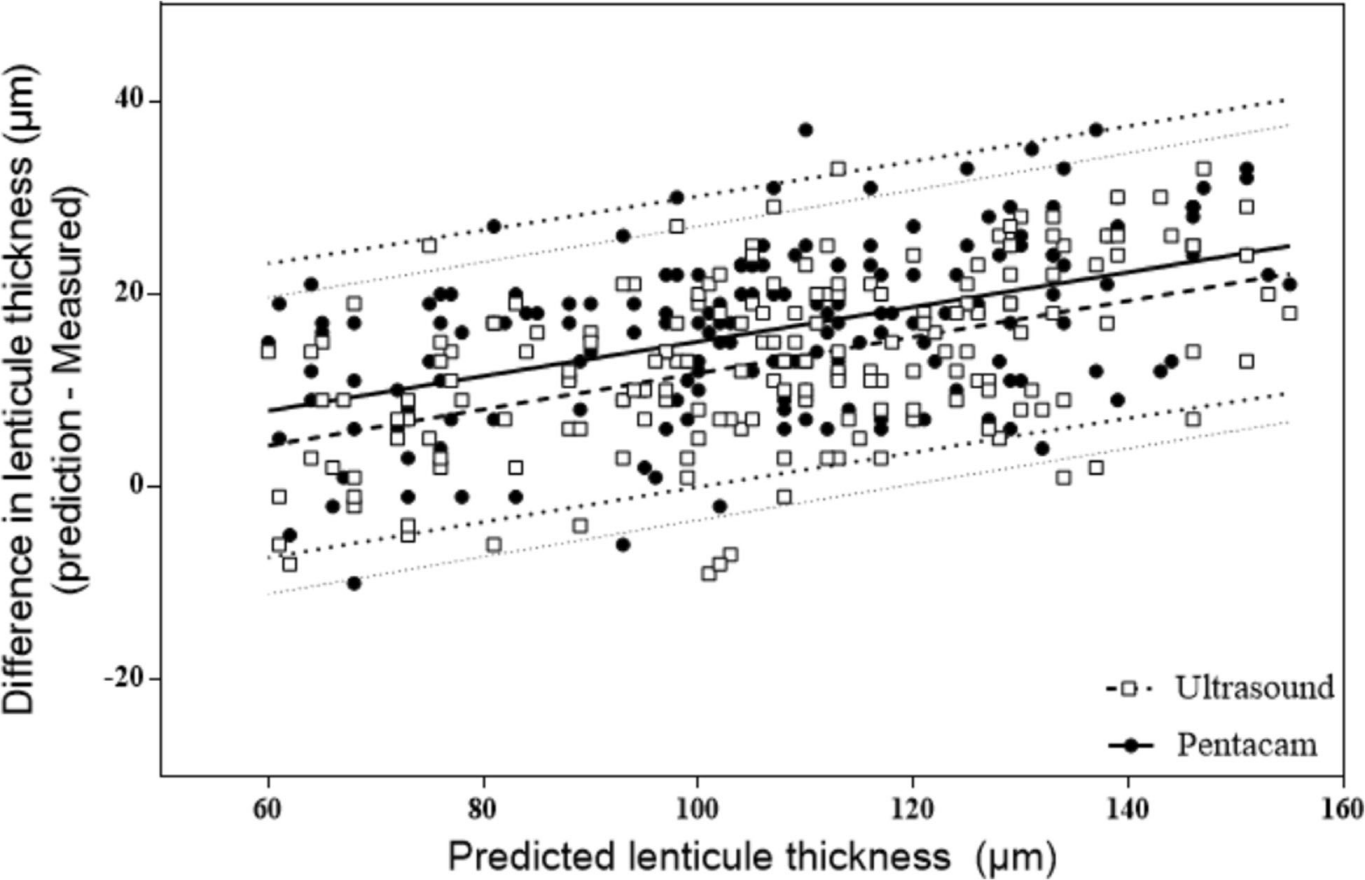
Regression-based 95% limits of agreement between predicted and measured lenticule thickness. The 95% limits of agreements (LoA) of the bias were calculated as the mean ± 1.96 standard deviations. Positive difference indicates an overestimation of the measured lenticule thickness. Bias ±95% limits of agreement are displayed (Table 2)

The preoperative and postoperative mean corneal pachymetry values are summarized in Table 1. No significant difference was detected between repeated measurements before and after surgery. Descriptive statistics of the VisuMax readout lenticule thickness, measured lenticule thicknesses, the difference between predicted and measured lenticule thickness are shown in the Table 2. No significant difference was found between the achieved lenticule thicknesses (ANOVA, *P* = 0.175). The VisuMax readout lenticule thickness was significantly greater than all of the achieved lenticule thicknesses measured with ultrasound pachymetry and Pentacam software (ANOVA, *P* < 0.001). On average, the VisuMax software prediction was found to overestimate the ultrasound-measured lenticule thickness by 13.02 μm and Pentacam-measured lenticule thickness by 16.26 at pupil center and 16.63 at cornea vertex. Linear regression detected significant relationships between the VisuMax readout lenticule thickness and all of the achieved lenticule thicknesses (Fig. 3). Figure 4 illustrates the 3-month ΔLT data points (ultrasound and pupil center from Pentacam) in the form of Bland-Altman plots (i.e., the ΔLT compared to the predicted lenticule thickness). We note that the mean of the ΔLT is significantly dependent on the magnitude of the measurement (ultrasound: *R*^2^ = 0.242, *P* < 0.001; pupil center from Pentacam: *R*^2^ = 0.230, *P* < 0.001).

**Table 1:** Mean preoperative and postoperative corneal pachymetry of subjects (μm)

Parameter	Preoperative	Postoperative
Ultrasound pachymetry	547.05 ± 27.34	453.52 ± 30.62
Corneal Vertex	545.46 ± 25.60	455.30 ± 29.70
Pupil Center	545.96 ± 25.63	455.38 ± 29.85
*P* Value^*^	0.736	0.798

Mean ± SD, *n* = 184; ^*^ ANOVA test.

**Table 2:** Characteristics of the lenticule thickness and ΔLT (μm)

Parameters	Predicted lenticule thickness	Measured lenticule thickness		
		Ultrasound	Pupil center	Corneal Vertex
Mean ± SD Range	106.55 ± 23.24 (60 ~ 155)	93.53 ± 20.39 (46 ~ 139)	90.30 ± 20.54 (42 ~ 134)	89.89 ± 20.47 (43 ~ 135)
Predicted to measured (ΔLT) (μm)	–	13.02 ± 8.84 (− 9 ~ 33)	16.26 ± 8.69 (− 10 ~ 37)	16.66 ± 8.87 (− 9 ~ 37)
95% limits of agreement	–	−4.67 ~ 30.71	− 1.11 ~ 33.63	− 1.07 ~ 34.39
Proportion of ΔLT in predicted value	–	11.9% ± 8.0%	15.1% ± 8.0%	15.4% ± 8.0%

For corneal pachymetry compared to ultrasound, a bias was shown by the Scheimpflug method (Table 3). In the present study, central corneal thickness before SMILE surgery was measured thinner by Scheimpflug imaging compared to ultrasound. The opposite tendency was obtained after SMILE surgery. The Bland-Altman analysis showed a tendency that the evident bias usually shows at extreme values of central corneal thickness (above 570 μm or under 420 μm) in Scheimpflug measurement comparisons with ultrasound pachymetry (Fig. 4).

**Table 3:** Pentacam measurements versus ultrasound pachymetry measurements (μm)

Parameters	Bias	SD	95% Limits of Agreement (μm)		
			Upper	Lower	Width
Preoperative corneal pachymetry					
Corneal Vertex	−1.59	7.80	13.70	−16.88	30.58
Pupil Center	−1.09	7.64	13.87	−16.06	29.93
Postoperative corneal pachymetry					
Corneal Vertex	2.07	6.46	14.73	−10.60	25.34
Pupil Center	2.15	6.51	14.91	−10.61	25.52

Difference (bias) in lenticule thickness measurements was calculated as predicted lenticule thickness minus measured lenticule thickness. (i.e., a positive difference indicated an overestimation of measured lenticule thickness). (Mean ± SD, *n* = 184). ΔLT: difference between predicted and achieved lenticule thickness.

Difference (bias) in corneal thickness measurements was calculated as Pentacam measurements minus ultrasound pachymetry (i.e., a negative difference indicated a thinner reading on Pentacam compared to ultrasound). (Mean ± SD, *n* = 184).

## Discussion

Myopia correction is accomplished through intrastromal lenticule extraction by femtosecond laser in SMILE surgery. Therefore, the predictability of lenticule thickness is the key to the accuracy of the SMILE procedure. The present study examined the relationship between the predicted and achieved lenticule thickness in eyes that had undergone SMILE for myopia and myopic astigmatism using the VisuMax femtosecond laser system.

**Fig. 5:**
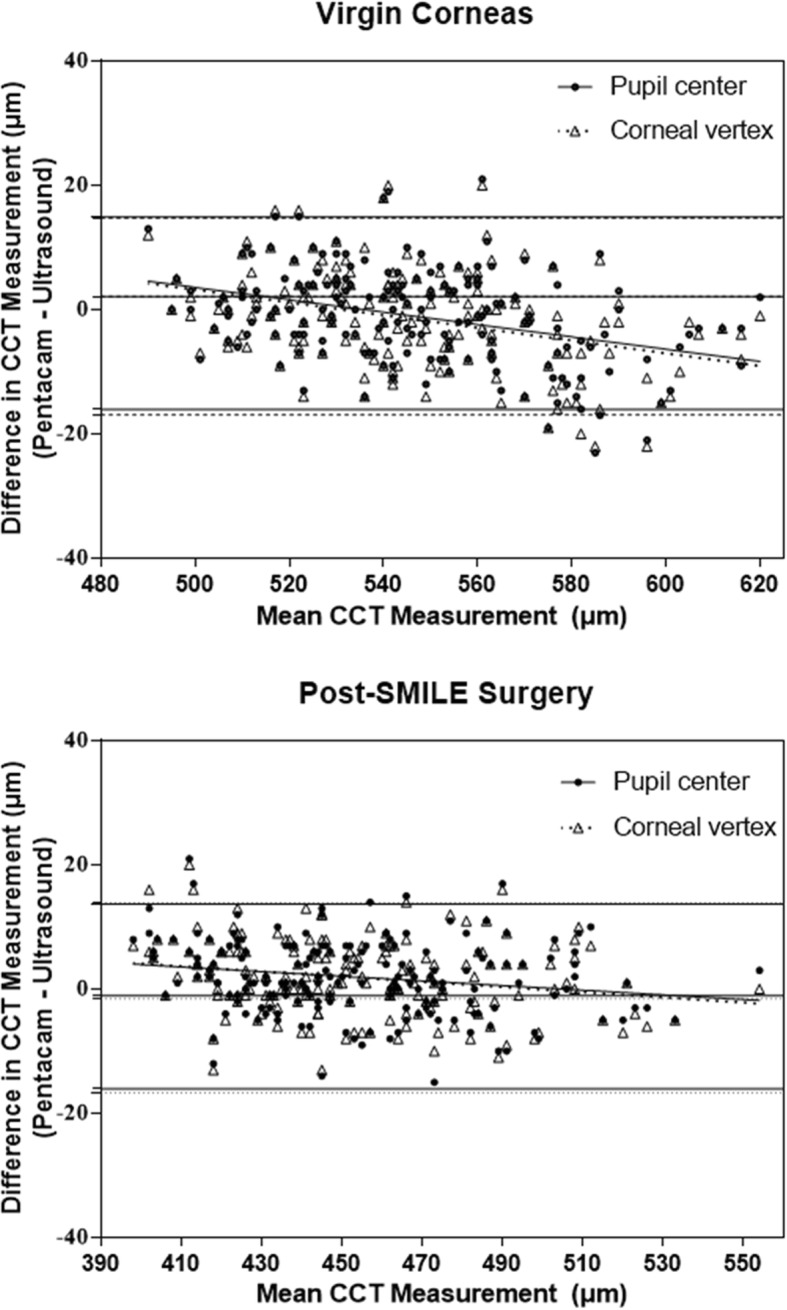
Bland–Altman charts displaying the difference between Scheimpflug Imaging and Ultrasound. In virgin and post-SMILE refractive surgery corneas, Bland–Altman charts displaying the difference for central corneal thickness measurements. The pupil center and corneal vertex were selected as the 2 locations for measurement calculation on Pentacam. A negative difference indicates a thinner reading on Pentacam compared to ultrasound. Bias ±95% limits of agreement are displayed (Table 3). (CCT: central corneal thickness)

For measurement of lenticule thickness, two techniques have been used in our studies, ultrasound pachymetry and Scheimpflug techniques. Ultrasound pachymetry has been the gold standard in measuring corneal thickness. The Bland-Altman analysis for the mean difference of lenticule thickness also indicates a close agreement between the ultrasonic-measured and Pentacam-measured lenticule thickness comparing with predicted lenticule thickness (Fig. 4). Considering the large population in this study, the Bland-Altman analysis (Fig. 5) showed a good agreement with the virgin cornea (95% LoA: − 16 to 14) and the post-SMILE cornea (95% LoA: − 11 to 15) when comparing the Scheimpflug techniques to ultrasound pachymetry (Table 3). On average, the Pentacam data showed a − 1.3 μm bias preoperatively and 2 μm bias postoperatively comparing with ultrasound pachymetry. The results suggest that the Pentacam overestimate in high value and underestimate in low value with respect to the ultrasound pachymetry. Similar results have been recently reported by peter et al. [20] (95% LoA: − 2.7 to 31.6).

In the present study, we found that the VisuMax readout was overestimated the achieved lenticule thickness (ultrasound) by a mean of 13.02 ± 8.87 μm (range: − 9 to + 33 μm). This was an anticipated finding, because Reinstein et al. [8] and Luft et al. [9] also note the stromal thickness reduction is overestimated by 8.2 μm and 9.8 μm, respectively (using very high-frequency-ultrasound or SD-OCT).

In the current study, significant relationships were found between the ΔLT (ultrasound and Pentacam) and the predicted lenticule thickness (Fig. 4). Our results agree with the results reported by Luft et al. [9] who reported theΔLT was significantly related to the preoperative SE. Liang et al. [13] also reported a significant difference in ΔLT between moderate (9.7 μm) and high (12.3 μm) myopia groups.

Previous studies have confirmed that VisuMax femtosecond laser has high accuracy in making LASIK flaps [21–23], so the systematic error in laser cutting can be excluded. Luft et al. and Reinstain et al. proposed that central stromal remodeling after SMILE might be the cause of the difference between predicted and measured lenticule thickness [8, 9]. The central stroma might expand after SMILE surgery as a result of tension release of the stromal collagen lamellae disrupted after the extraction of the lenticule. The finding in the present study may agree with this theory. Considering that thicker lenticule extraction may result in more tension release, the central stroma may expand more and increase the ΔLT.

The overestimation of the achieved lenticule thickness in the SMILE procedure indicates that the postoperative stromal reduction was less than expected. In the present study, the proportion of ΔLT (overestimation) in predicted value is 11.9% for ultrasound and about 15% for Pentacam. We believed that the detected difference between predicted and measured lenticule thickness may be the cause of the difference between the treated SE with nomogram adjustment (− 5.85 ± 1.79 D) and the achieved SE (− 5.36 ± 1.61 D). For each patient, a 10% correction of spherical correction was added to the nomogram to compensate for the loss of postoperative stromal reduction, which was also close to 10%. At 3 months, the mean postoperative SE was 0.02 ± 0.17 D (range: − 0.75 to 0.5 D). Only 14% of patients showed slight postoperative hyperopia between 0.14 D to 0.50 D (Fig. 2). The results demonstrate that the change in the nomogram is appropriate.

A personal nomogram for SMILE surgery was also found in previous studies. Liang et al. [13] suggested adding 11% correction of SE to the nomogram for SMILE surgery. Zhou et al. [14] adjusted the mean treated SE up to − 6.30 ± 2.00 D when the mean preoperative SE was − 5.96 ± 1.97 D in SMILE surgery. Reinstein et al. [8] reported that a mean under correction of − 0.78 D would be expected if he did not change the nomogram. In the current study, the nomogram adds 10% correction of spherical refractive to achieve emmetropia.

The overestimation of achieved lenticule thickness may exclude eligible SMILE patient. Our results suggest that when screening SMILE patients, clinicians should subtract 10% of the predicted lenticule thickness to calculate the residual corneal stroma bed thickness.

## Conclusions

In conclusion, an overestimation of the achieved lenticule thickness was evident in this study. The ΔLT which is significantly related with the predicted lenticule thickness might be the cause of the difference between the treated SE and achieved SE. Also, our results showed that 10% increase of spherical refractive correction in the nomogram is appropriate. Furthermore, clinicians should subtract 10% of the predicted lenticule thickness to calculate the residual corneal stroma bed thickness.

## Data Availability

The datasets used and/or analyzed during the current study are available from the corresponding author on reasonable request.

## Abbreviations

ANOVA: Analysis of variance
D: Diopters
LoA: Limits of agreements
MRSE: Manifest refraction spherical equivalent
PTA: Percent tissue alert
SE: Spherical equivalent
SD: Standard deviation
SMILE: Small incision lenticule extraction
UDVA: Uncorrected distance visual acuity
ΔLT: The differences between the predicted and achieved lenticule thickness.
